# Effectiveness of biologic switching in a real‐life, Belgian severe asthma cohort

**DOI:** 10.1002/iid3.70023

**Published:** 2024-10-08

**Authors:** Lucie Lafarge, Charles Pilette, Céline Bugli, Antoine Froidure

**Affiliations:** ^1^ Pneumology Department Cliniques Universitaires Saint‐Luc, Université catholique de Louvain Brussels Belgium; ^2^ Pole of Pulmonology, ENT and skin (LUNS Lab) Institut de Recherche Expérimentale et Clinique, UCLouvain Louvain‐la‐Neuve Walloon Brabant Belgium; ^3^ Support en Méthodologie et Calcul Statistique (SMCS) Université catholique de Louvain Brussels Belgium

## Abstract

In this observational study, we show that switching biologics in severe asthma results in a high proportion of controlled patients.

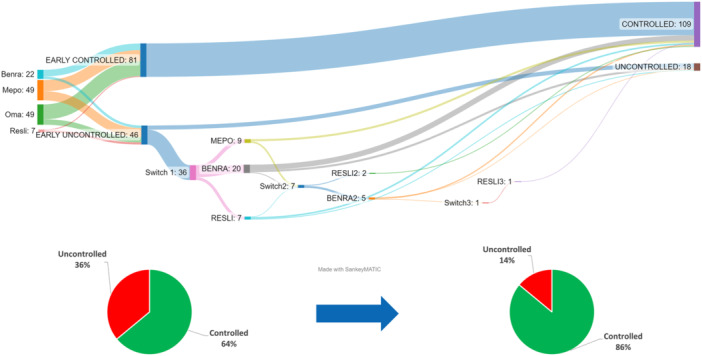

## 1 BACKGROUND

Despite appropriate phenotyping, one‐fourth^1^ of severe asthma (SA) patients are nonresponders to a first biologic, leading to persistent exacerbations and impaired quality of life. Although common in clinical practice, real‐life effectiveness of switching is poorly explored. Therefore, the characteristics of switchers in our center was explored.

## 2 METHODS

We conducted a retrospective, monocentric, observational cohort study. We included all adult patients who were diagnosed with SA according to ATS‐ERS guidelines^2^ from 2006 to 2023 and who received at least one biologic. We compared nonswitchers (one biologic received) and switchers (at least two biologics received) and studied type of switches and their effectiveness. We defined responders as patients who experienced a 50% reduction in oral corticosteroids or more and/or a 50% reduction of exacerbations or more, associated with a 3‐point improvement (minimal clinically important difference) in Asthma Control Test (ACT) and, when relevant (i.e., in case of rhinosinusitis with nasal polyps [CRSwNP].), a subjective improvement of ear, nose, and throat (ENT) symptoms over a minimum follow‐up period of 6 months. Both groups were compared with respect to their demographic characteristics, their asthma phenotype (age of onset, atopic status, and comorbidities), the levels of type 2 biomarkers (blood eosinophils, exhaled fraction of nitric oxide—FeNO) and the use of inhaled and/or oral corticosteroids.

All data were pseudonymised for manipulation. Values are expressed as median and range for continuous variables and percentages for categorical variables. Kruskal−Wallis test was used for continuous variables and Pearson's chi‐squared test for categorical variables; a *p* < .05 was considered significant. Statistics were performed by our Faculty statistics platform (statistical methodology and computing service *UCLouvain*). The study was approved by our internal UCLouvain review board (ref. 2006‐121).

## 3 RESULTS

We included 127 patients. The first biologic was omalizumab for 49 patients, mepolizumab for 49 patients, benralizumab for 22 patients and reslizumab for seven patients. We identified 36 switchers (28%) for a total of 44 switches. Table 1 provides the main characteristics of our cohort. We did not find any difference regarding sex ratio, smoking status, blood eosinophils, allergic status or level of FeNO. However, as compared to nonswitchers, switchers had an earlier onset of the disease (47 vs. 55 years‐old, *p* = .02) and had more frequently ENT comorbidity including CRSwNP (58% in switchers vs. 38% in nonswitchers, *p* = .03).

**Table 1:** characteristics of switchers and nonswitchers before the initiation of biologics.

		Nonswitchers (*n* = 91)	Switchers (*n* = 36)	*p* Value
Male, *n* (%)		44 (48)	15 (42)	.50
Diagnostic age of asthma (year)		34 (1−72)	31 (1−69)	.53
Diagnostic age of severe asthma (year)		55 (4−82)	47 (4−75)	.02
BMI (kg/m^2^)		26 (16−38)	25 (18−47)	.14
Smoking status	Never smoked, %	59 (65)	23 (64)	.33
	Ex‐smoker, %	24 (26)	8 (22)	
	Current smoker, %	5 (5)	1 (3)	
Exacerbations, *n* over last 12 months		3 (0−15)	3 (0−12)	.93
ACT score (pts)		12 (5−25)	11 (5−25)	.44
Blood eosinophils (/µL)		530 (0−3720)	630 (0−4040)	.30
Atopic status, *n* (%)		60 (77)	25 (76)	.89
IgE (kU/L)		209 (7−7490)	192 (24−4231)	.93
FeNO (ppb)		36 (0−234)	46 (2−221)	.32
ENT involvement	None, %	20 (22)	2 (6)	**.03**
	Non allergic rhinitis, *n* (%)	3 (3)	1 (3)	.88
	Allergic rhinitis, *n* (%)	18 (20)	3 (8)	.11
	Chronic rhinosinusitis without nasosinusal polyposis, *n* (%)	11 (12)	5 (14)	.78
	Chronic rhinosinusitis with nasosinusal polyposis, *n* (%)	34 (38)	21 (58)	.03
Treatment	Inhaled beclometasone equivalent (µg)	1600 (100−4000)	2000 (400−4000)	.57
	Systemic corticosteroids, *n* (%)	28 (31)	12 (33)	.96
	Prednisolone equivalent dose (mg/day)	9 (2.5−40)	10 (2.5−20)	.22
Initial response rate		81 (89)	4 (11)	

*Note*: Characteristics of switchers (*n* = 36) and nonswitchers (*n* = 91).

Abbreviations: ACT, asthma control test; BMI, body mass index; ENT, ear, nose and throat; FeNO, fractional exhaled nitric oxide.

**Figure 1:**
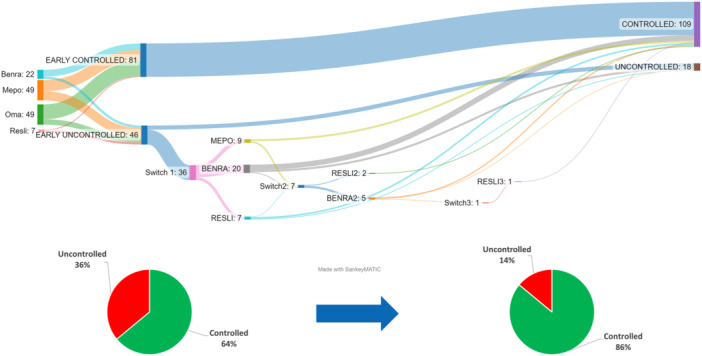
Sequence of biologics in 127 patients with severe asthma; showing the switch sequence and clinical response for all the patients over time.

The sequences of switches are shown in Figure 1: Seven patients performed a second switch (20%) and one had a third switch and thus received all biologics available in Belgium at that time. The majority of switches were between anti‐IL5 and anti‐IL5R (34/44, 77%). Ten switches (23%) were from anti‐IgE to anti‐IL5/IL5R. No patient switched from anti‐IL5/IL5R to omalizumab. Reasons for switching were poor asthma (*n* = 25), poor ENT control (*n* = 32) and side effects or change in the administration route (from intraveinous reslizumab to subcutaneous anti‐IL5/R) despite asthma control (*n* = 4).

Seventy‐five percent of the uncontrolled patients benefited from the switch(es). After their last switch, the annual severe exacerbation rate decreased from 2.3 ± 2.09 to 0.82 ± 0.98, with 66% of patients having *a* ≥ 50% reduction of their exacerbation rate. ACT improved from 14.1 ± 5.8 to 18.5 ± 5.2, with 63% of patients improving ≥ 3 points. Furthermore, 9 out of the 13 cortico‐dependent patients (69%) reduced the oral corticosteroids daily dose by ≥50%. Of note, 10 patients (8%) among the nonswitchers finally stopped the biotherapy due to lack of efficacy without having benefited from a switch.

## 4 DISCUSSION

Switching biologics in SA is common, yet past studies mostly analyzed single switches between two biologics. Our study enabled us to address multiple switches in real‐life and within the same class of anti‐IL‐5/IL‐5R antibodies. Clinical characteristics between switchers and nonswitchers appeared relatively similar, making the identification of predictive features challenging. Nevertheless, a notably higher frequency of nasal polyps was observed among switchers, consistently with previous studies.^3^ Additionally, switchers exhibited a younger age at diagnosis, a distinguishing feature consistent with findings in Matsumoto‐Sasaki's study.^3^ Female gender and the presence of aspirin‐exacerbated respiratory disease were also more prevalent among switchers in this study. In a recent study,^4^ higher blood eosinophil counts and FeNO levels were detected among switchers.

Reasons for switching are primarily inadequate asthma control or ENT symptoms, in line with previous studies, emphasizing persistent asthma symptoms as the main driver.^5^ Efficacy of switching varies across studies, from 32% to 90%, depending on the criteria used.^1^, ^5^, ^6^, ^7^, ^8^ In the OSMO study, 77% of patients switching from omalizumab to mepolizumab had a significant improvement in the ACQ‐5 score and 50% displayed an increased FEV1.^6^ Another study demonstrated improvements in quality of life, exacerbation rate and corticosteroid use after switching from mepolizumab to benralizumab or reslizumab.^7^ Of course, our study comes with some limitations, starting with its single‐center and retrospective aspects. Nevertheless, observed switch efficacy in our cohort is similar to other multicentric cohorts. Another aspect is that it only included anti‐IgE and anti‐IL‐5 (R) compounds. Newer biologics Dupilumab and Tezepelumab, targeting IL‐4/IL‐13 and TSLP, respectively, unavailable until late 2023 in Belgium have also demonstrated efficacy in SA patients in terms of exacerbation reduction, symptoms control and lung function improvement.^8^ Access to a larger panel of SA medication further highlights the need for careful phenotyping of patients to maximize the initial response. In conclusion, this observational study shows that an earlier onset of SA and chronic rhinosinusitis with nasal polyps are associated with an increased prevalence of switch in biologic‐treated SA patients. With regard to switches between anti‐IgE and anti‐IL5 (R), this clinical practice appears effective to achieve disease control in most patients (75%). Future studies with detailed phenotyping and biomarkers should help to better predict the effectiveness of switching biologics in SA.

## AUTHOR CONTRIBUTIONS

**Lucie Lafarge**: Conceptualization. **Charles Pilette**: Conceptualization. **Céline Bugli**: Conceptualization.

## CONFLICT OF INTEREST STATEMENT

Charles Pilette discloses unrestricted research grants from GlaxoSmithKline, Chiesi, Sanofi and AstraZeneca, outside of the submitted work. Antoine Froidure discloses unrestricted research grants from Boehringer Ingelheim, outside of the submitted work. The remaining authors declare no conflict of interest.

## CLINICAL IMPLICATIONS BOX

This study shows an effectiveness of 75% of biological switches in asthma, with a proportion of patients of 64% controlled after a first biotherapy rising to 86% after one or several switch(es).

## Supporting information

Supporting information.
